# ﻿*Phyllodiaptomusparachristineae*, a new species of copepod (Copepoda, Calanoida, Diaptomidae) from the floodplain of the lower Mekong River Basin in Thailand and Cambodia

**DOI:** 10.3897/zookeys.1168.104636

**Published:** 2023-07-04

**Authors:** Laorsri Sanoamuang, Santi Watiroyram

**Affiliations:** 1 Applied Taxonomic Research Center, Faculty of Science, Khon Kaen University, Khon Kaen 40002, Thailand; 2 International College, Khon Kaen University, Khon Kaen 40002, Thailand; 3 Division of Biology, Faculty of Science, Nakhon Phanom University, Nakhon Phanom 48000, Thailand

**Keywords:** Freshwater, Mun River, *P.christineae*, Southeast Asia, taxonomy, Tonle Sap Lake

## Abstract

Phyllodiaptomus (Phyllodiaptomus) parachristineae**sp. nov.**, a new diaptomid copepod, was collected from 30 sites in the lower Mekong River Basin floodplain in northeastern Thailand and nine sites in Cambodia. The new species is the 13^th^ species of the genus to have been recorded across Asia. It has a wide range of habitats, occurring in small to large, temporary to permanent water bodies, and it can be found at any time of the year. The morphology of the new species is most similar to that of the males of P. (P.) christineae, having a comb on the antepenultimate segment on the right antennule, symmetrical caudal rami, a narrow hyaline lamella on the left P5 basis, a distal accessory spine on the right P5Exp-2, and a single-lobed Enp on the right P5. On the other hand, the males of the new species have second and third urosomites without hair-like setae on the ventral margin, a rounded distal margin of the P5 intersclerite plate, a semi-circular lamella on the right P5 basis, a strong principal lateral spine inserted at the proximal 1/3 of the right P5Exp-2, and a two-segmented left P5Enp. Morphological differences among species in the subgenus Phyllodiaptomus (Phyllodiaptomus) as well as the distribution and habitats of the 13 species and two subspecies of *Phyllodiaptomus* in Asia are discussed.

## ﻿Introduction

The Mekong River is Southeast Asia’s longest and the 12^th^ longest river in the world. It flows 4,350 kilometers from the Tibetan Plateau to the Mekong Delta and into the South China Sea, making it one of Asia’s major river systems (Sanoamuang and Watiroyram 2018). Approximately 75% of the Mekong’s drainage area is located in Laos, Thailand, Cambodia, and Vietnam. The Mekong River catchment area is divided into two major sections. The upper part of the Mekong flows 1,955 kilometers through southwestern China, accounting for approximately 25% of the total land area. The lower part of the Mekong is a 2,390 km-long stretch that drains the Khorat Plateau of northeastern Thailand, the western slopes of the Annamese Cordillera in Laos and Vietnam, and the majority of Cambodia before reaching the sea in a form of delta in southern Vietnam (White et al. 2023).

The Mekong River basin is one of the most biodiverse regions in the world, with more than 20,000 plant species and 850 fish species identified there. Approximately 80% of the nearly 65 million inhabitants who live in the lower Mekong River basin make their living from the river and its abundant natural resources (MRC 2023). The Mun River, the longest Mekong tributary, flows through northeastern Thailand for 641 kilometers. In 1994, 265 fish species were recorded in its watershed (Jutagate et al. 2003).

The Tonle Sap Lake and the Mekong River dominate the landscape of Cambodia. During the dry season, the 120-km-long Tonle Sap River, a tributary of the Mekong, connects Tonle Sap Lake to the Mekong near Phnom Penh. The tropical monsoon has a significant influence on this unique and intricate hydrological system. This relatively flat-bottomed freshwater lake is quite shallow during the dry season and rarely exceeds 3.3 meters in depth. During the rainy season, the lake can reach a depth of 8–10 meters. During the rainy season (mid-May to early October), the Mekong River floods, causing water to back up into the Tonle Sap River and flow into Tonle Sap Lake. Flooding and reverse flows extend the dry-season lake (120 km long by 35 km wide) into its floodplain, forming a wet-season lake that is 250 km long and 100 km wide (Olson and Morton 2018).

Copepods of the genus *Phyllodiaptomus* Kiefer, 1936, are known to inhabit freshwater habitats in Asia. Thirteen species are currently identified in the countries of central, eastern, western, southern, and southeast Asia (Zhang et al. 2023). Dumont et al. (1996) classified the genus into two subgenera. Four species have been recognized as belonging to the subgenus Phyllodiaptomus (Ctenodiaptomus) Dumont, Ranga Reddy & Sanoamuang, 1996: *P.annae* (Apstein, 1907) from Sri Lanka; *P.sasikumari* Ranga Reddy & Venkateswarlu, 1989 from India; *P.wellekensae* Dumont & Ranga Reddy, 1993 from India; and *P.praedictus* Dumont & Ranga Reddy, 1994 from Thailand. There are nine species in the subgenus Phyllodiaptomus (Phyllodiaptomus) Dumont, Ranga Reddy and Sanoamuang (1996):

1. *P.blanci* (Guerne & Richard, 1896) from Uzbekistan;
2. *P.tunguidus* Shen & Tai, 1964 from China, Laos and Vietnam;
3. *P.longipes* Kiefer, 1965 from Indonesia;
4. *P.irakiensis* Khalaf, 2008 from Iraq;
5. *P.christineae* Dumont, Ranga Reddy & Sanoamuang, 1996 from Thailand;
6. *P.surinensis* Sanoamuang & Yindee, 2001 from Thailand;
7. *P.thailandicus* Sanoamuang & Teeramaethee, 2006 from Thailand;
8. *P.roietensis* Sanoamuang & Watiroyram, 2020 from Thailand and Cambodia; and
9. *Phyllodiaptomus* sp. from Thailand (Sanoamuang & Dabseepai, 2021) and Cambodia (Chaicharoen & Sanoamuang, 2022).

Here, we describe the previously called *Phyllodiaptomus* sp. from Thailand and Cambodia as *Phyllodiaptomusparachristineae* sp. nov. The new species have been found at several sites in the floodplains of the Mekong River tributaries, the Mun River in Thailand, and the Tonle Sap Lake-River Complex in Cambodia. It is the sixth species in Thailand, the third in Cambodia, and the 13^th^ in the genus.

## ﻿Materials and methods

**Figure 1.**
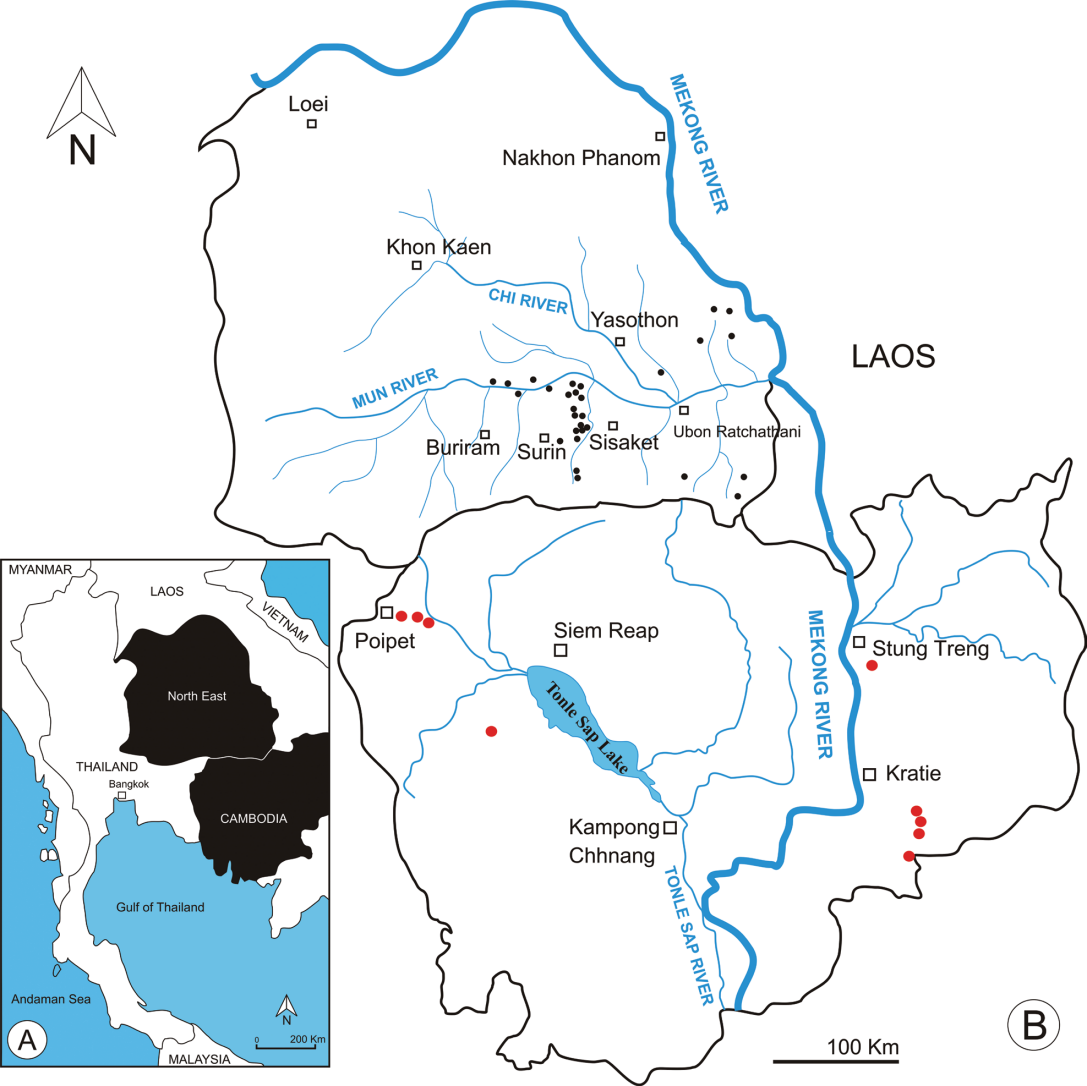
Sampling sites of Phyllodiaptomus (P.) parachristineae sp. nov. in Thailand (black dots) and Cambodia (red dots). Open black squares indicate cities.

In Thailand, samples were qualitatively taken from the Mun River Basin in Surin Province (December 1998, August 1999, and April 1999) and Ubon Ratchatani Province (June 2002 and October 2002) (Fig. 1). In total, 379 samples were obtained from 224 sites (temporary and permanent waters), including rice fields, roadside canals, ponds, and reservoirs. In Cambodia, samples were collected from 252 sites in seven provinces (Banteay Meanchey, Battambang, Siem Reap, Kampong Thom, Pursat, Kratie, and Stung Treng) from February to October 2007.

Samples were collected using a 60-µm-mesh plankton net and preserved immediately in 4% formalin. Adult specimens were sorted under an Olympus SZ51 stereomicroscope at 40-x magnification and placed in a mixture of glycerol and 70% ethanol (ratio of 1:10 v/v). The animals were then transferred to a small drop of pure glycerol. They were dissected and prepared on a glycerin-mounted slide under a stereomicroscope at 40–100× magnification. All appendages and body ornamentation were examined at 1000× magnification under an Olympus CX31 compound microscope. The drawings were made using an Olympus U-DA drawing tube mounted on a compound microscope. The final versions of the drawings were made using the CorelDRAW 12.0 graphic program.

The dissected specimens were prepared for semi-permanent slides by mounting them in pure glycerine and sealing them with transparent nail varnish. All un-dissected specimens were stored in 70% ethanol in 1.5 mL microtubes. Type specimens are deposited at the Thailand Natural History Museum, Pathum Thani, Thailand (**THNHM**), and the Applied Taxonomic Research Center, Khon Kaen University, Khon Kaen, Thailand (**KKU**).

Abbreviations used in this paper are as follows: **ae**, aesthetasc; **Enp**, endopod; **Exp**, exopod; **Exp/Enp-n**, exopodal segment, n/endopodal segment n; **P1–P5**, legs 1–5; **sp**, spine.

## ﻿Taxonomic section

**Order Calanoida Sars, 1903**

**Infraorder Neocopepoda Huys & Boxshall, 1991**

**Family Diaptomidae Baird, 1850**

**Sub-family Diaptominae Kiefer, 1932**

﻿**Genus *Phyllodiaptomus* Kiefer, 1936**

**SubgenusPhyllodiaptomus Dumont, Ranga Reddy & Sanoamuang, 1996**

### ﻿ Phyllodiaptomus (P.) parachristineae sp. nov.

Taxon classificationAnimaliaCalanoidaDiaptomidae

3A68C4E1-B26E-5BCE-AC70-E837068CB27C

https://zoobank.org/C90C4B7C-1CD5-422B-9E7D-8D5F567F482B

**Figure 2.**
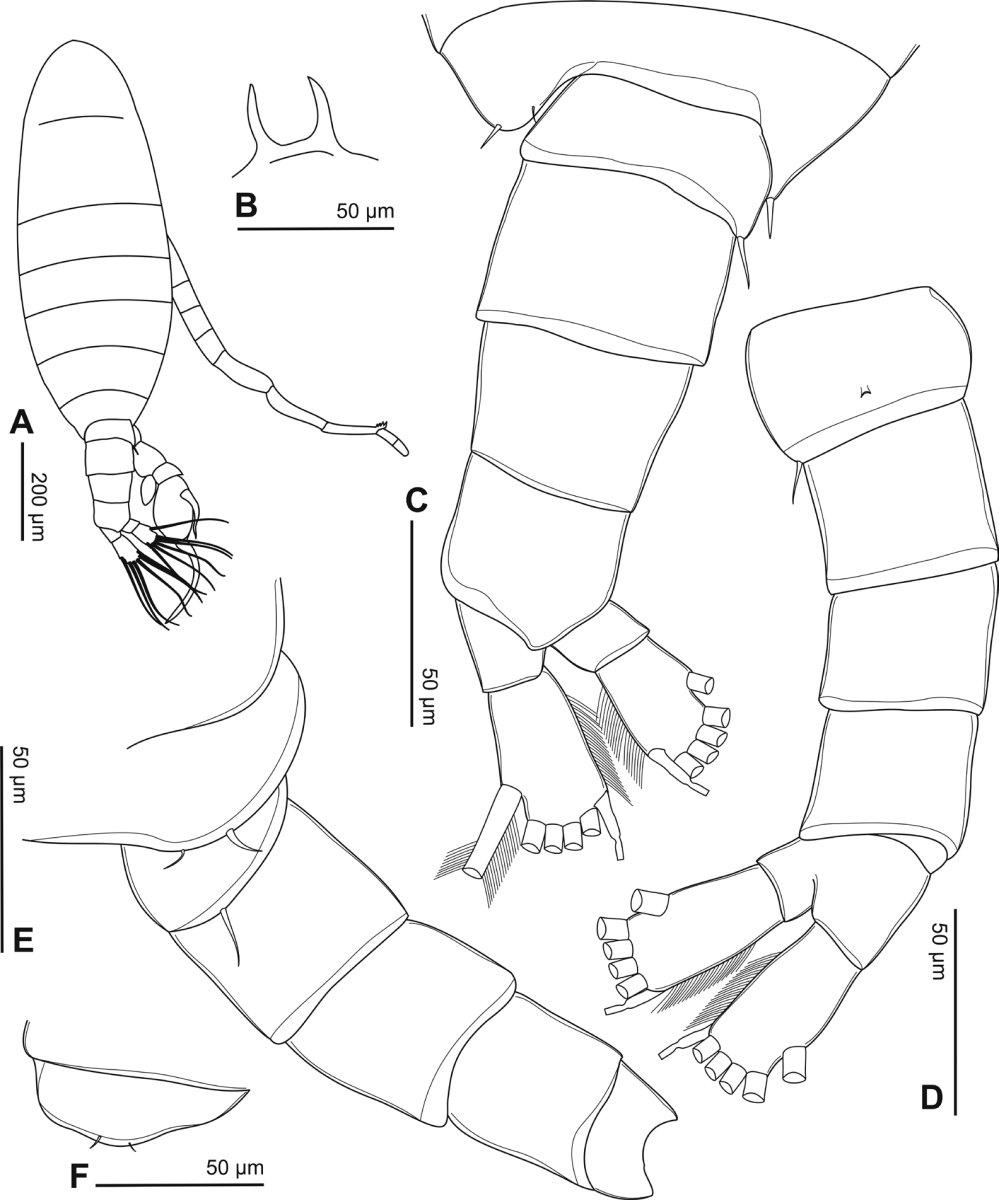
Phyllodiaptomus (P.) parachristineae sp. nov., male: **A** habitus, dorsal view **B** rostrum **C** prosomite 5 and urosome, dorsal view **D** urosome, ventral view **E** prosomite 5 and urosome, right side in lateral view **F** left wing of prosomite 5.

**Figure 3.**
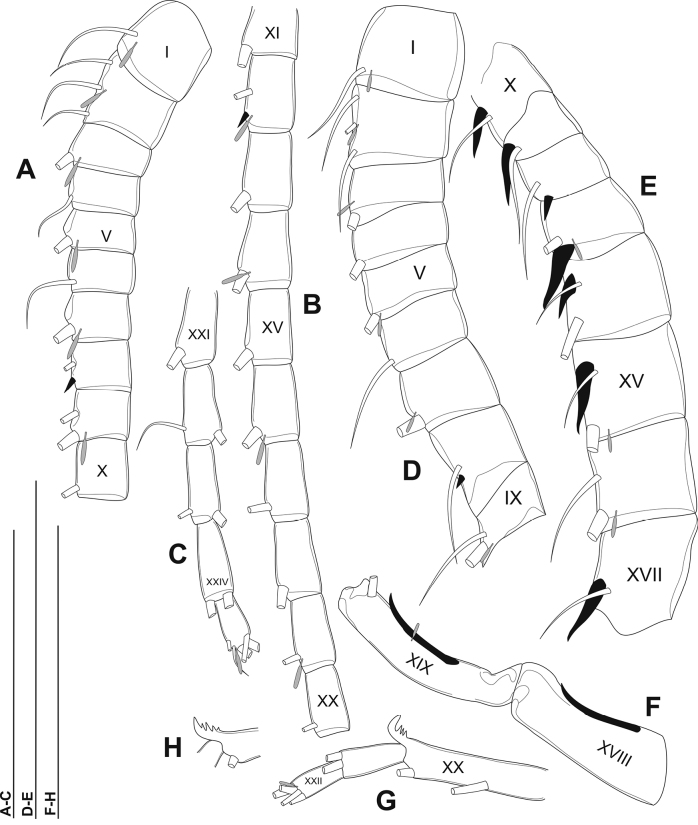
Phyllodiaptomus (P.) parachristineaesp. nov., male, antennule: **A–C** left antennule **A** segments I–X **B** segments XI–XX **C** segments XXI–XXV **D–H** left antennule **D** segments I–IX9 **E** segments X–XVII **F** segments XVIII–XIX **G** segments XX–XXII **H** a variation of segment XX.

**Figure 4.**
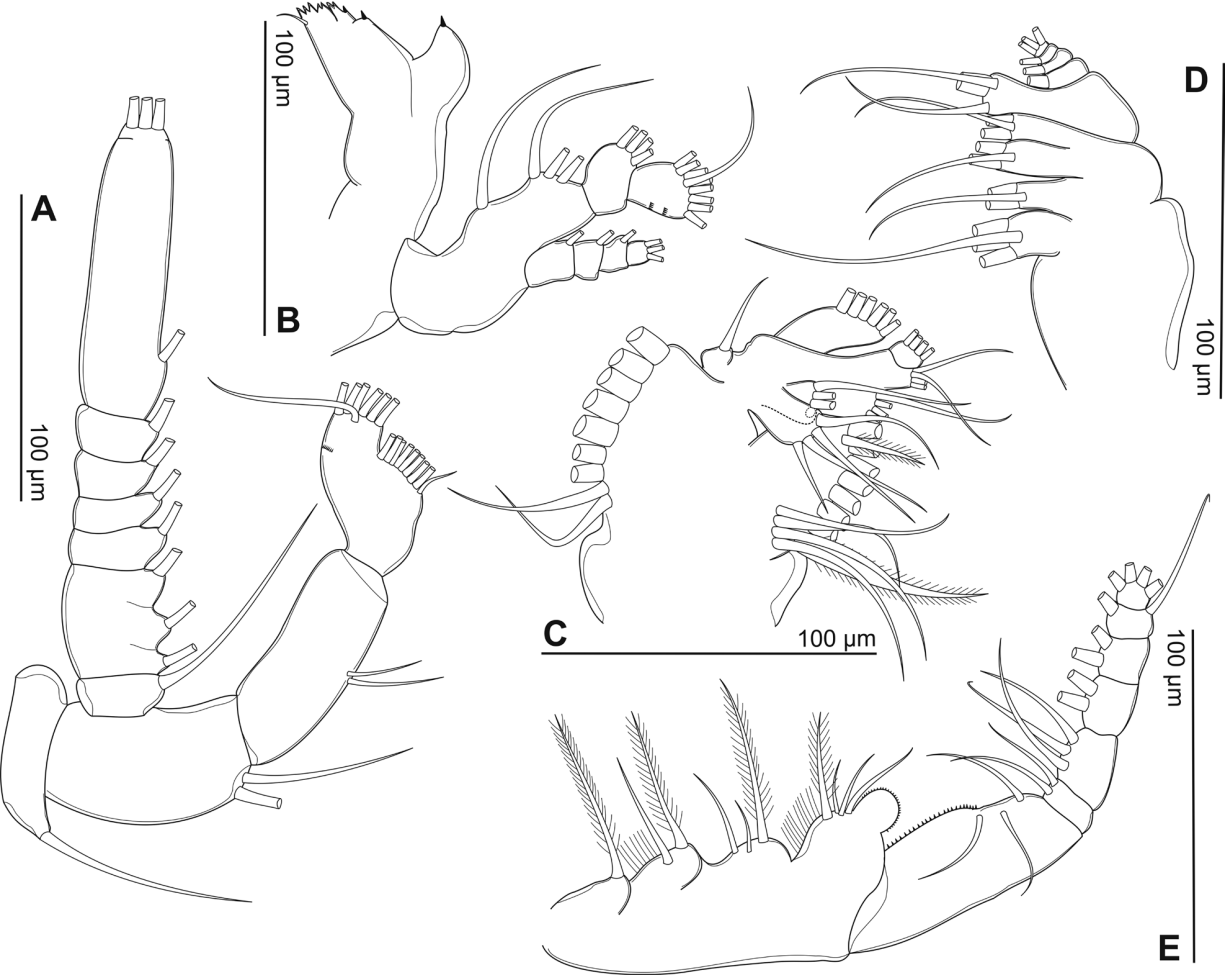
Phyllodiaptomus (P.) parachristineaesp. nov., male: **A** antenna **B** mandible **C** maxillule **D** maxilla **E** maxilliped.

**Figure 5.**
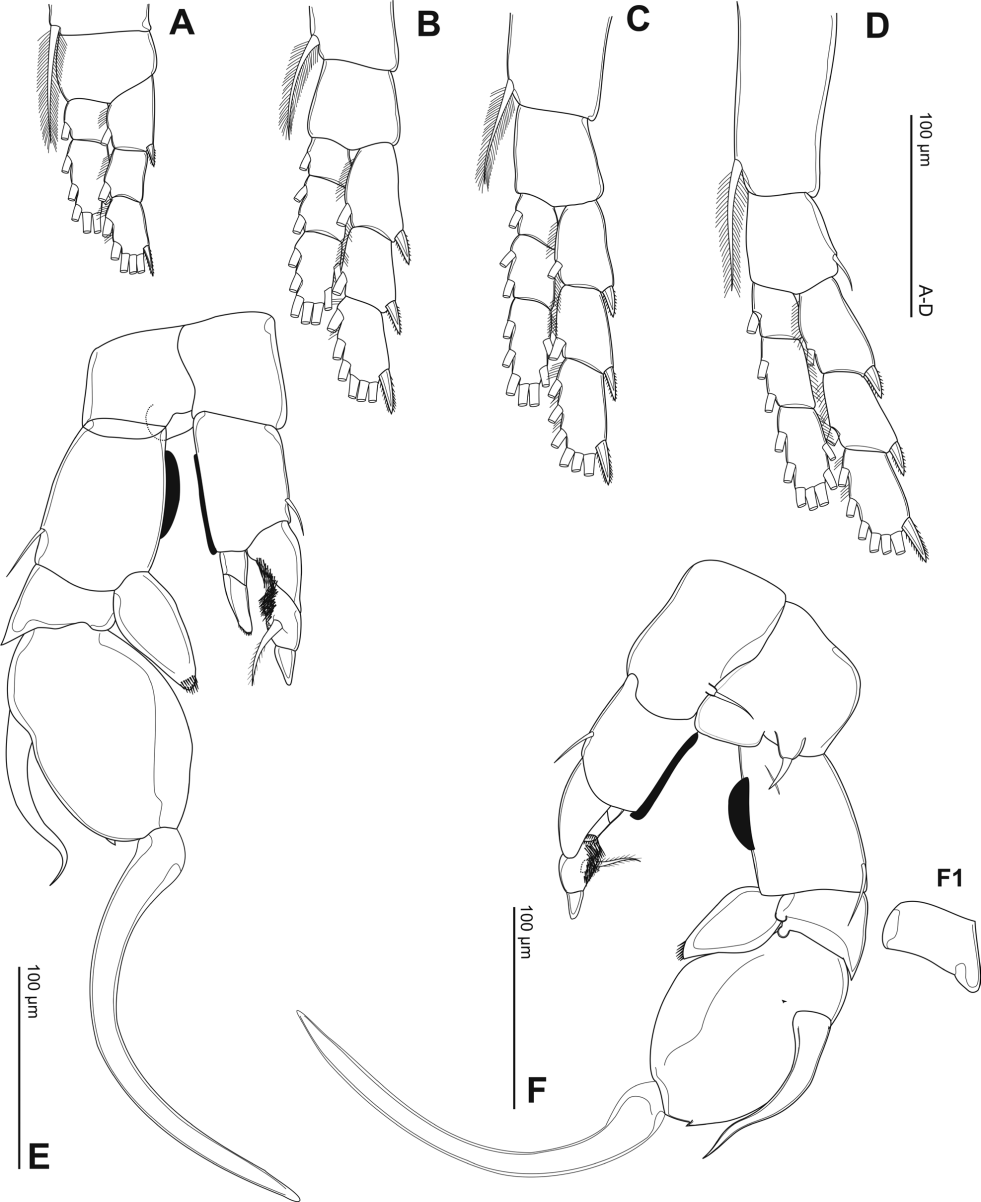
Phyllodiaptomus (P.) parachristineaesp. nov., male: **A**P1**B** P2 **C** P3 **D** P4 **E**P5 in anterior view **F**P5 in posterior view **F1** a variation of right P5Exp-1.

**Figure 6.**
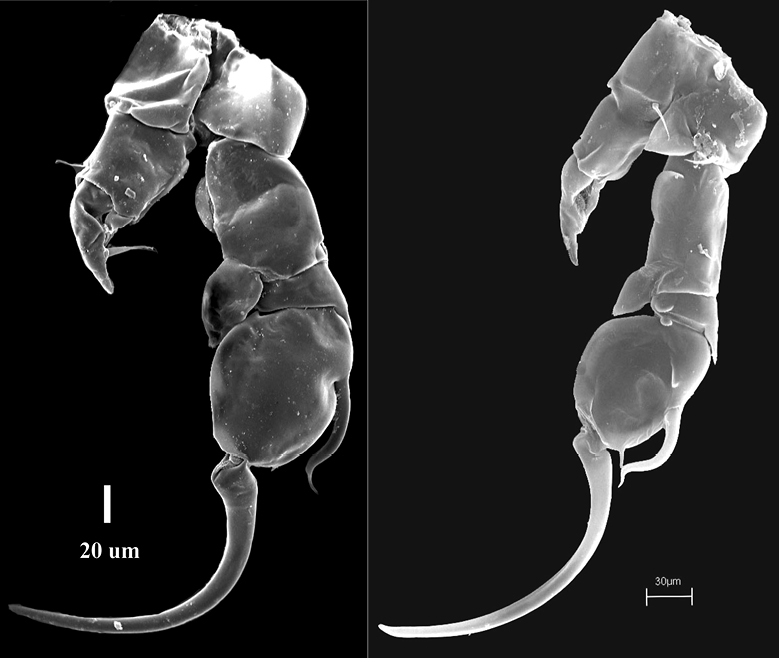
SEM pictures of the male’s P5 of Phyllodiaptomus (P.) parachristineaesp. nov. (**A**) and P. (P.) christineae (**B**; fig. 2B in Sanoamuang and Dabseepai 2021, p. 9) (posterior view).

**Figure 7.**
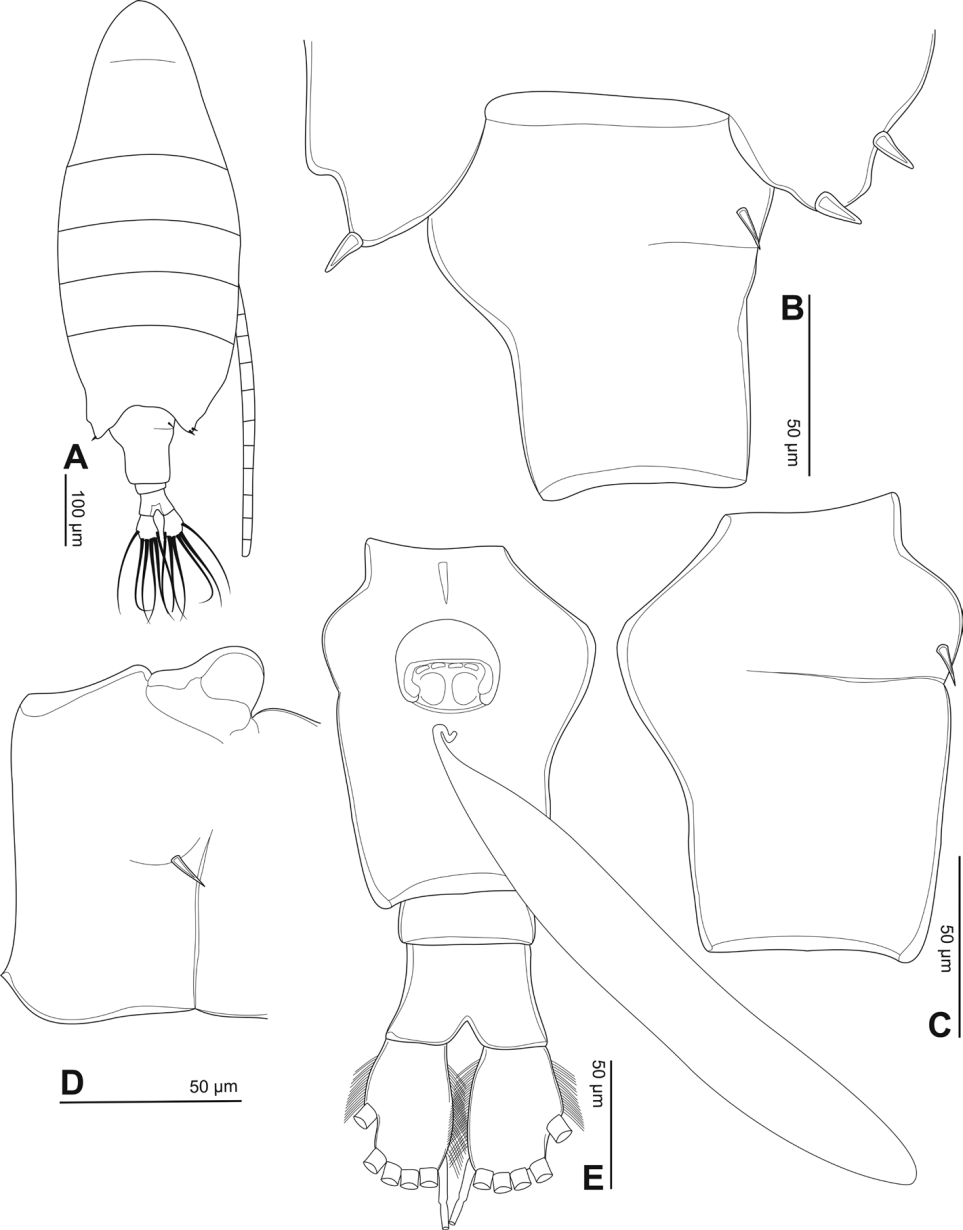
Phyllodiaptomus (P.) parachristineaesp. nov., female: **A** habitus, dorsal view **B** prosomite 5 and genital double-somite, dorsal view **C** genital double-somite, dorsal view **D** right spine on genital double-somite, lateral view **E** urosome with spermatophore, ventral view.

**Figure 8.**
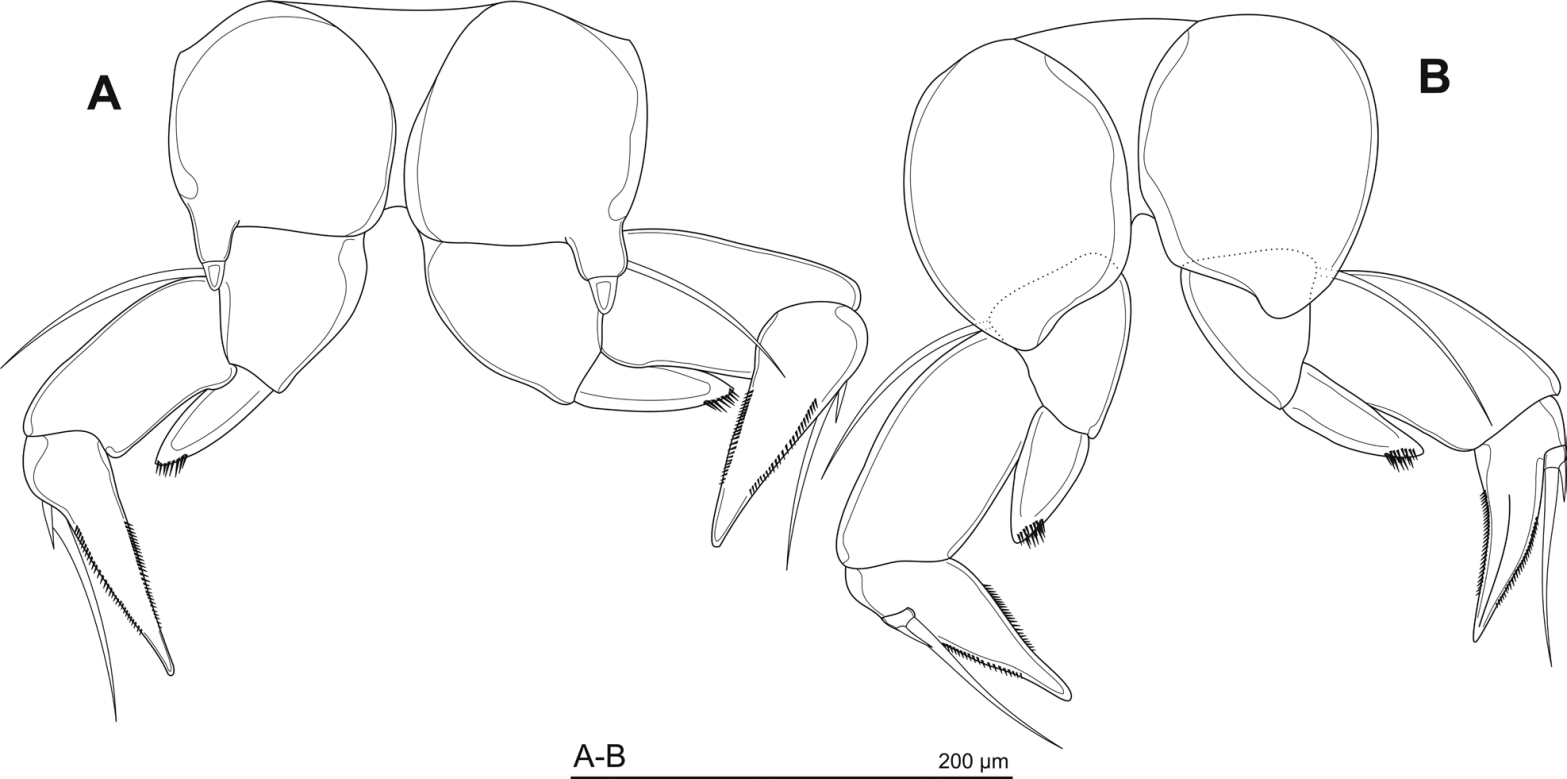
Phyllodiaptomus (P.) parachristineaesp. nov., female: **A**P5, posterior view **B**P5, anterior view.

Figs 2 , 3 , 4 , 5 , 6 , 7 , 8

Phyllodiaptomus (P.) sp.: Sanoamuang and Dabseepai (2021): 7, 19, 22.
Phyllodiaptomus (P.) sp.: Chaicharoen and Sanoamuang (2022): 1, 6–7, 9, 11–12; fig. 2u.

#### Type locality.

A temporary pond, near Km 6 of the road no. 2214, Nong Om Subdistrict, Thung Si Udom District, Ubon Ratchathani Province, northeastern Thailand (14°49'14"N, 104°59'09"E); water temperature 28.3 °C, pH 6.8, conductivity 67 µS cm^-1^.

#### Type material.

***Holotype***: one adult male completely dissected (THNHM-IV-20183, one slide), collected from the type locality on 11 June 2002, by L. Sanoamuang. ***Allotype***: one adult female, completely dissected (THNHM-IV-20184, one slide). ***Paratypes***: one adult female and three adult males, undissected (THNHM-IV-20185–20186); two adult females and five adult males, undissected (KKU-COP-2019-T-01), preserved in 70% ethanol; collected from the type locality on the same date as the holotype.

#### Other localities.

**Thailand**: eight temporary-water bodies from Ubon Ratchathani Province, northeast Thailand, sampled by P. Wansuang: (1) a temporary pond, Ban Don Yoong, Muang Sam Sip Subdistrict, Muang Sam Sip District, collected on 19 October 2002; (2, 3) roadside canals, along the road no. 2050 at Kms 25 and 43, Hua Na Subdistrict, Khemarat District, collected on 9 June 2002; (4) a roadside canal, along the road no. 2337 at Km 4, Pho Sai Subdistrict, Pho Sai District, collected on 9 June 2002; (5) a roadside canal, along the road no. 2248 at Km 94, Huai Kha Subdistrict, Buntharik District, collected on 10 June 2002; (6) a rice field, road no. 2248 at Km 65, Na Chaluai Subdistrict, Na Chaluai District, collected on 10 June 2002; (7) a temporary pond, along the road no. 2214 at Km 7, Khok Chamrae Subdistrict, Thung Si Udom District, collected on 11 June 2002; and (8) a temporary pond, Ban Na Sabaeng, Ka Bin Subdistrict, Kut Khaopun District, collected on 20 October 2002.

Twenty-one localities from Surin Province, northeast Thailand, were sampled by W. Yindee:

1. a roadside canal, Ban Doo Sok, Nong Ha Subdistrict, Samrong Thap District, collected on 7 December 1998;
2. a canal, Ban Nong Ha, Nong Ha Subdistrict, Samrong Thap District, collected on 7 December 1998;
3. a roadside canal, Ban Khon Kaen, Nong Ha Subdistrict, Samrong Thap District, collected on 7 December 1998;
4. a permanent pond, Ban Khon Kaen, Nong Ha Subdistrict, Samrong Thap District, collected on 7 December 1998;
5. a canal, Ban Nong Buaban, Nong Buaban Subdistrict, Rattanaburi District, collected on 7 December 1998;
6. a canal, Ban Chat, Nong Buaban Subdistrict, Rattanaburi District, collected on 7 December 1998;
7. a permanent pond, Ban Rawiang, Rawiang Subdistrict, Non Narai District, collected on 7 December 1998;
8. a canal, Ban Non, Don Rat Subdistrict, Rattanaburi District, collected on 7 December 1998;
9. a rice field, Ban Nonsadao, Nong Thap Subdistrict, Non Narai District, collected on 7 December 1998;
10. a temporary pond close to the Mun river, Tha Tum Subdistrict, Tha Tum District, collected on 9 December 1998;
11. a reservoir, Ban Nongbor, Ba Subdistrict, Tha Tum District, collected on 9 December 1998;
12. a canal, Ban Tato, Phrom Thep Subdistrict, Tha Tum District, collected on 9 December 1998;
13. a canal, Ban Phrai Khla, Phrai Khla Subdistrict, Chumphon Buri District, collected on 9 December 1998;
14. a canal, Ban Sri Chumpon, Chumpon Buri Subdistrict, Chumphon Buri District, collected on 9 December 1998;
15. a canal, Ban Kut Phatai, Kut Wai Subdistrict, Sikhoraphum District, collected on 10 December 1998;
16. a permanent pond, Ban Narong, Narong Subdistrict, Si Narong District, collected on 10 December 1998;
17. a reservoir, Ban Angkhor, Ban Chan Subdistrict, Sangkha District, collected on 10 December 1998;
18. Huay Saneng Dam, Chaniang Subdistrict, Mueang District, collected on 10 December 1998;
19. a canal, Ban Nayom, Khaen Subdistrict, Sanom District, collected on 11 December 1998;
20. a roadside canal, Sanom intersection, Sanom Subdistrict, Sanom District, collected on 11 December 1998; and
21. a roadside canal, Taphet intersection, Na Nuan Subdistrict, Sanom District, collected on 7 December 1998.

**Cambodia**: Nine localities were sampled by R. Chaichareon:

1. a canal, Mong Rusey, preytouch, Dub Krasaing, Battambong Province (12°49'52"N, 103°24'22"E), collected on 9 June 2007;
2. a canal, Ochrov, Nimith, Acphiwatt, Bantaen Meanchey Province (13°37'15"N, 102°42'58"E), collected on 9 June 2007;
3. a ricefield, Srey Sophol, Teuk Thla, Teuk Thla, Bantaen Meanchey Province (13°34'55"N, 102°53'05"E), collected on 9 June 2007;
4. a canal, Srey Sophol, Teuk Thla, Teuk Thla, Bantaen Meanchey Province (13°34'46"N, 102°54'49"E), collected on 9 June 2007;
5. a temporary pond, Strung Treng District, Strung Treng Province (13°17'11"N, 106°06'20"E), collected on 10 June 2007;
6. a permanent pod, Snourl, Sreicha, Meanchey, Kratie Province (11°58'48"N, 106°23'16"E), collected on 22 October 2007;
7. a swamp, Snourl, Snourl, Prek Kdey, Kratie Province (13°08'09"N, 106°26'19"E), collected on 22 October 2007;
8. a permanent pond, Snourl, Ksen, Kratie Province (12°13'08"N, 106°26'07"E), collected on 22 October 2007; and
9. a canal, Kratie District, Kratie Province (12°15'57"N, 106°25'42"E), collected on 22 October 2007.

#### Diagnosis.

**Male.** Right antennule with a serrated spine on segment XX. Urosomites 2 and 3 without hairs ventrally. Caudal rami symmetrical. P5: intersclerite plate produced into a round lobe distally. Right P5: coxa with moderate spine on posterior lobe; right basis with semi-circular lamella on inner medial margin; Exp-1 with outer distal margin produced into acute tip; Exp-2 spherical to oval with principal lateral spine inserted at 1/3 length of outer margin; tiny distal accessory spine; Enp one-segmented. Left P5: basis with long, narrow lamella; Exp-2 with spinular field on inner margin; Enp two-segmented.

**Female.** Pedigerous somites 4 and 5 completely fused. Pedigerous somite 5 with asymmetrical wings: right wing round with two strong spines, left wing triangular with one strong spine. Genital double-somite incompletely fused dorsolaterally, with right dorsolateral spine. Exp-2 without lateral spine.

#### Description of adult male.

Body length, without caudal setae, 0.9–1.1 mm (mean = 1.0 mm, *n* = 5; Fig. 2A). Prosome ovoid, ~ 2.5× as long as urosome (Fig. 2A). Rostrum with bifid process on distal margin (Fig. 2B). Cephalosome with transversal groove dorsally at anterior part of somite. Pediger 4 separated dorso-laterally from pediger 5 (Fig. 2E). Pediger 5 asymmetrical; right postero-lateral wing triangular in dorsal view but round on the left wing; posterior spines larger than dorsal spine; spines on right wing relatively larger than those on the left wing (Fig. 2E, F).

Urosome (Fig. 2C–E) with five somites, unornamented. Genital somite dilated postero-laterally on right side, shorter than wide, with a minute spine on posterolateral corner. Urosomites 2–4 as long as wide. Anal somite asymmetrical, twisted to right side (Fig. 2C). Caudal rami symmetrical, each ramus 2.5× as long as wide, inner margin hairy (Fig. 2C, D). Each ramus with six setae, subequal in length and size, plumose: dorsal seta bare and thinner than others.

Antennule (Fig. 3): asymmetrical, not reaching beyond the end of caudal setae. Left antennule (Fig. 3A–C): 25-segmented. Armature formula as in Table 1.

**Table 1.** Armature formula of the left male antennule of Phyllodiaptomus (P.) parachristineaesp. nov. The number of setae (Arabic numerals), aesthetascs (ae), and spines (sp) is given. The Roman numerals refer to segment numbers.

	**Segment number**												
	**I**	**II**	**III**	**IV**	**V**	**VI**	**VII**	**VIII**	**IX**	**X**	**XI**	**XII**	**XIII**
Number of armature	1+ae	3+ae	1+ae	1	1+ae	1	1+ae	1+sp	2+ae	1	1	1+ae+sp	1
	**XIV**	**XV**	**XVI**	**XVII**	**XVIII**	**XIX**	**XX**	**XXI**	**XXII**	**XXIII**	**XXIV**	**XXV**	
Number of armature	1+ae	1	1+ae	1	1	1+ae	1	1	2	2	2	5+ae	

Right antennule (Fig. 3D–H) 22-segmented. Armature formula as in Table 2. Segment XX (antepenultimate) with spine modified into comb-like (4 teeth) process (Fig. 3H).

**Table 2.** Armature formula of the right male antennule of Phyllodiaptomus (P.) parachristineaesp. nov. The number of setae (Arabic numerals), aesthetascs (ae), and spines (sp) is given. The Roman numerals refer to segment numbers.

	**Segment number**										
	**I**	**II**	**III**	**IV**	**V**	**VI**	**VII**	**VIII**	**IX**	**X**	**XI**
Number of armature	1+ae	3+ae	1+ae	1	1+ae	1	1+ae	1+sp	2+ae	1+sp	1+sp
	**XII**	**XIII**	**XIV**	**XV**	**XVI**	**XVII**	**XVIII**	**XIX**	**XX**	**XXI**	**XXII**
Number of armature	1+sp	1+ae+sp	2+sp	2+ae+sp	2+ae	1+sp	sp	1+ae+sp	2+sp	2	4+ae

Antenna (Fig. 4A): coxa and basis with one and two bare setae on inner distal corner, respectively. Enp two-segmented. Enp-1 with two setae along inner margin; Enp-2 with nine setae along inner margin, seven setae apically; all setae bare. Exp seven-segmented: Exp-1–6 with 1, 3, 1, 1, 1, 1 setae along inner margin; Exp-7 with one seta on inner margin and three setae apically; all setae bare.

Mandible (Fig. 4B): ca 7 cuspidate teeth dorsally and three small triangular teeth on cutting blade ventrally; one bare seta on coxal gnathobase dorsally. Basis with four bare setae: one proximally and three distally along inner margin. Enp-1 with four setae on inner distal corner. Enp-2 with nine setae apically; two oblique rows of spinules along outer margin. Exp-1–3 each with one seta on inner margin; Exp-4 with three setae apically; all setae bare.

Maxillule (Fig. 4C): praecoxal arthrite with nine strong setae laterally and four slender submarginal setae. Coxal endite with four setae; coxal epipodite with nine setae; two proximal-most setae smaller than others. Two basal endites fused to segment bearing them: proximal and distal endite, each with four setae apically; basal exite with one short seta. Enp-1 and Enp-2 each with four setae apically, proximal segment fused to basis. Exp with six setae apically.

Maxilla (Fig. 4D): praecoxa fused to coxa. Proximal and distal endites on praecoxa with four and three setae apically, respectively. Two coxal endites with three setae apically each. Allobasis with three setae apically. Enp four-segmented: Enp-1–3 with one inner seta each, Enp-4 with three setae apically.

Maxilliped (Fig. 4E): four medial lobes on syncoxa: setal formula 1, 2, 3, 4, respectively; subdistal inner margin produced into a spherical lobe ornamented with densely tiny spinules. Basis with three setae along medial margin, with a row of tiny spinules proximately. Enp-1–6 with 2, 3, 2, 2, 2, and 4 setae, respectively.

P1–P4 (Fig. 5A–D): each with a round and bare intercoxal sclerite. All coxae with bi-pinnate setae on inner distal margin. P1–P3 basis without setae; a reduced bare seta on outer margin of P4. Exp longer than Enp; two-segmented Enp and three-segmented Exp on P1, three-segmented Enp and Exp on P2–P4. Armature formula of P1–P4 as in Table 3.

**Table 3.** Armature formula of the swimming legs of Phyllodiaptomus (P.) parachristineaesp. nov. The number of setae (Arabic numerals) and spines (Roman numerals) is given in the following sequence: outer-inner margin or outer-apical-inner margin.

	Coxa	Basis	Exp			Enp		
			1	2	3	1	2	3
P1	0-1	0-0	I-1	0-1	I-3-2	0-1	1-2-3	–
P2	0-1	0-0	I-1	I-1	I-3-3	0-1	0-2	2-2-3
P3	0-1	0-0	I-1	I-1	I-3-3	0-1	0-2	2-2-3
P4	0-1	1-0	I-1	I-1	I-3-3	0-1	0-2	2-2-3

P5 (Figs 5E, F, 6A): intercoxal sclerite fused to coxa, produced into a rounded lobe distally (Fig. 5F1). Right P5: coxa with an acute, strong spine on posterior surface. Basis subrectangular, larger than that on the left side; semi-circular lamella at the middle of inner margin; short longitudinal ridge at proximal half of posterior surface; short, thin seta on the distal outer margin. Enp one-segmented, distal half tapering, tipped with tiny spinules; reaching beyond 1/3 of Exp-2, close to insertion of its principal lateral spine on outer margin. Exp-1 shorter than wide, with a bifid knob at inner margin; distolateral margin with a small acute process. Exp-2 oval with two lateral spines. Principal lateral spine articulated, located at 1/3 length of Exp-2 outer margin, thick, S-shaped, with a sharp curved tip, reaching the distal margin of segment. Accessory lateral spine minute, close to insertion of end-claw. End-claw sickle-shaped, with a blunt tip; ~ 1.5× as long as Exp-2.

Left P5 (Figs 5E, F, 6A): coxa with thin seta on posterior lobe near distal inner corner; longer, slender than spine on right coxal segment. Basis with narrow hyaline lamella along 3/4 distal length of inner margin; with a small seta at distal outer margin. Exp-1 triangular, tapering towards distal end, medial margin concave with a field of long setules. Exp-2 smaller than Exp-1, bulbous, with a strong pinnate seta at mid-length of medial margin, with a field of long setules proximally, and a field of tiny spinules distally. Apical hyaline process, thumb-like, with blunt tip. Enp two-segmented, shorter than Exp-1; Enp-1 unarmed. Enp-2 longer than Enp-1, slightly tapering distal end, tipped with a row of tiny spinules.

#### Description of adult female.

Body length, without caudal setae, 0.9–1.2 mm (mean = 1.1 mm, *n* = 5), slightly larger than male (Fig. 7A). Prosome: urosome ratio ~ 2.4:1. Prosome similar to that of males, but lateral wings on pedigerous somites 4‒5 completely fused. Lateral wings on pediger 5 asymmetrical: round on right side, triangular on left side; right wing with two strong spines; left wing with one spine (Fig. 7B). Urosome 3-segmented, with asymmetrical genital double-somite (Fig. 7A, 7E). Genital double-somite longer than urosomite 2, anal somite, and caudal rami combined, but incompletely fused dorsolaterally on right side (Fig. 7B, C, E). Right side with a small spine at proximal 1/2 of segment. Left side with bulged margin proximally compared to right side. A pair of gonopores and copulatory pores located centrally at ~ 1/2 length of genital double-somite, beneath a genital operculum. Urosomite 2 symmetrical, shorter than wide. Anal somite (Fig. 7A, E) as long as wide; anal operculum small with convex free margin.

Antennule symmetrical; left antennule, antenna, mouthparts, and P1–P4 as in male.

P5 (Fig. 8) asymmetrical. Coxa with a blunt, stout spine on distal outer margin. Basis with a thin, bare seta on outer margin, reaching almost distal end of Exp-1. Exp-1 sub-rectangular, more than twice as long as wide, longer than Enp. Exp-2 triangular, with a row of strong spinules along both margins; right side stouter than left one (Fig. 8A); left side with a longitudinal groove (i.e., a conveyor canal) on anterior view (Fig. 8B). Exp-3 reduced, represented by a small segment on proximal outer margin of Exp-2, with one short spine and a longer medial spiniform seta apically. Enp one-segmented, conical, with a circular row of spinules apically.

#### Variation.

The male specimens of the new species have the P5 variations as follows: (1) right P5Exp-1: distal outer corner shape varies from triangular to round (Fig. 5F1); (2) right P5Exp-2: differences in size of a large distal outer spine; (3) left P5Enp: varies in length (Figs 5E, F); (4) the comb-like process on segment XX of the right antennule has 4–5 teeth (Fig. 3H).

#### Etymology.

The specific name *parachristineae* is a combination of the Greek prefix *para*-, meaning to resemble, and the specific name *christineae*, referring to the fact that the male P5 of the new species resembles Phyllodiaptomus (P.) christineae Dumont, Ranga Reddy & Sanoamuang, 1996 (Fig. 6B).

#### Distribution.

At present, the new species have been found throughout the year in both temporary and permanent water bodies, like ponds, roadside canals, irrigation canals, puddles in rice fields, and reservoirs. The new species was prominently found at 30 sites in the Mun River Basin, which is one of the largest tributaries of the Mekong River in Thailand (Fig. 1). In Cambodia, the new species has been found in habitats similar to those in Thailand. However, it was recorded at only nine of the 252 sampled sites in the floodplain of Tonle Sap Lake and the Mekong River Basin (Fig. 1).

Temperatures in the waters where the new species was discovered range from 20.0–34.1 °C, pH 6.0–7.9, and conductivity 39.4–810.0 µS cm^-1^. The new species was always present with other diaptomid species, ranging from 2–6 species per sampled site. Co-occurrences of diaptomid species were *Dentodiaptomusjavanus* (Grochmalicki, 1915), *Eodiaptomusphuphanensis* Sanoamuang, 2001a, *Mongolodiaptomusbotulifer* (Kiefer, 1974), *M.malaindosinensis* (Lai & Fernando, 1978), *M.dumonti* Sanoamuang, 2001b, *M.mekongensis* Sanoamuang & Watiroyram, 2018, *Neodiaptomuslaii* Kiefer, 1974, *N.yangtsekiangensis* Mashiko, 1951, Phyllodiaptomus (P.) surinensis Sanoamuang & Yindee, 2001, *Tropodiaptomusoryzanus* Kiefer, 1937, *T.vicinus* (Kiefer, 1930), and *Vietodiaptomusblachei* (Brehm, 1951). The co-occurrence of congeneric species was rare, with only one site with the new species and P. (P.) surinensis living together.

## ﻿Discussion

The new species belongs to the subgenus Phyllodiaptomus (Phyllodiaptomus) Dumont, Ranga Reddy & Sanoamuang, 1996, and its congeners are P. (P.) blanci (Guerne & Richard, 1896), P. (P.) christineae, P. (P.) irakiensis Khalaf, 2008, P. (P.) longipes Kiefer, 1965, P. (P.) roietensis, P. (P.) surinensis, P. (P.) thailandicus, and P. (P.) tunguidus Shen & Tai, 1964. They obviously differ from the subgenus Phyllodiaptomus (Ctenodiaptomus) Dumont, Ranga Reddy & Sanoamuang, 1996, by the fact that the male P5 has the left Exp-2 with a patch of strong spinules along the medial margin, versus the members of the subgenus Phyllodiaptomus (Ctenodiaptomus) having spinular outgrowth that is like a comb-shaped fan.

Phyllodiaptomus (P.) parachristineaesp. nov. can be easily distinguished from the three other species of the subgenus Phyllodiaptomus (Phyllodiaptomus) in Thailand by morphological differences in the males. The new species has an unproduced distal margin of the intersclerite plate, whereas P. (P.) thailandicus has a distinct, bilobed one. The new species has a moderate spine on the right coxa, versus a large spine in P. (P.) roietensis and P. (P.) surinensis, but a reduced spine in P. (P.) thailandicus. The new species has no chitinous process on the right basis, but it is present in these three species. The right P5 of the new species has a single-lobed Enp, but a bilobed Enp in P. (P.) roietensis and P. (P.) surinensis. The principal lateral spine of new species is inserted on the proximal half of its Exp-2 outer margin, but such a spine is inserted on the middle length of Exp-2 in P. (P.) thailandicus and on the distal half in P. (P.) roietensis and P. (P.) surinensis. The new species has symmetrical caudal rami, but they are transformed in P. (P.) roietensis and P. (P.) surinensis. Furthermore, the new species lacks hairs on urosomites 2–3 ventrally, whereas P. (P.) thailandicus has hairy urosomites 2 and 3 (for more information, see Table 4).

**Table 4.** Morphological differences among species in the subgenus Phyllodiaptomus (Phyllodiaptomus).

Characters	Species								
	P. (P.) blanci	P. (P.) christineae	P. (P.) irakiensis	P. (P.) longipes	P. (P.) roietensis	P. (P.) surinensis	P. (P.) thailandicus	P. (P.) tunguidus	P. (P.) parachristineaesp. nov.
**Male**									
P5 intersclerite plate	Triangular lobed	Mostly triangular lobed	Bi-lobed	Triangular lobed^1^	Unproduced (shallow lobed)	Unproduced (shallow lobed)	Bi-lobed	Conical	Rounded lobed
Right P5 coxa	Moderate spine	Moderate spine	Moderate spine	Moderate spine	Large spine	Large spine	Reduced spine	Moderate spine	Moderate spine
Right P5 basis	With small round lamella	With narrow lamella	With small amorphous lamella	With narrow lamella	With chitinous process, triangular lamella	With chitinous process, bi-lobed lamella	With chitinous	With narrow lamella, chitinous process	With semi-circular lamella
Right P5Enp	Single-lobed	Single-lobed	Single-lobed	Single-lobed	Bi-lobed	Bi-lobed	Single-lobed	Single-lobed	Single-lobed
Right P5Exp-2	Without accessory spines	With distal accessory spine	With distal accessory spine (on hyaline lobe)	With distal accessory spine	With three accessory spines	With three accessory spines	Without accessory spines	Without accessory spines	With reduced distal accessory spine (mostly)
Principal spine on right P5Exp-2	At distal half, sturdy	At middle, slender	At middle, sturdy	At proximal half, sturdy	At distal half, sturdy	At distal half, sturdy	At middle, sturdy	At distal half, slender	At proximal half, sturdy
Left P5 basis	With small lamella	With narrow lamella	Without lamella^2^	Without lamella	With narrow lamella	Without lamella, with two small spines	With narrow lamella	With narrow lamella	With narrow lamella
Lelf P5Enp	Unsegmented	Unsegmented	Two-segmented	Unsegmented	Two-segmented	Unsegmented	Unsegmented	Two-segmented	Two-segmented
Right caudal ramus	Normal	Normal	Normal	Normal	Transformed	Transformed	Normal	Transformed	Normal
Urosome	Urosomite 2 hairy	Urosomite 2 hairy	Unornamented	Urosomites 2–3 hairy	Unornamented	Unornamented	Urosomites 2–3 hairy	Unornamented	Unornamented
Right antennule segment 20	Serrated	Serrated	Serrated	Smooth	Serrated	Serrated	Serrated	Serrated	Serrated
**Female**									
Genital double-somite	With a pair of spines	With a pair of spines	With a pair of spines	With a pair of spines	With a pair of spines	With a pair of spines, with ventral hyaline outgrowth	With a pair of spines	With a pair of spines	With a right spine
P5	N/A	Asymmetrical (Elongate Exp-Enp)	Symmetrical	N/A	Asymmetrical	Symmetrical	Symmetrical	Asymmetrical	Asymmetrical
P5Exp-2	With seta	With seta	With seta	With seta	With seta	With seta	With seta	With seta	Unarmed
Conveyor canal on P5Exp-2	Present	Present^3^	N/A	N/A	Present	Present	Present	Present	Present
P5 Enp	Two-segmented	Two-segmented	Two-segmented	Unsegmented	Two-segmented	Two-segmented	Two-segmented	Two-segmented	Two-segmented

^1^ = Kiefer (1965) has drawn the distal inner margin of the intercoxal sclerite with a roughly triangular lobe rather than a conical lobe in the view of Dumont and Ranga Reddy (1993). ^2^ = Khalaf (2008) described the left P5 basis of a male with a small hyaline lobe projecting from the distal inner corner, but such a hyaline did not figure on their leg. ^3^ = Dumont et al. (1996) mentioned that the conveyor canal in the SEM figure is not clear.

The male of the new species is also different from the males of the four other Asian species in that the distal edge of the P5 interslerite plate is rounded instead of bilobed, conical, or triangular, as it is in P. (P.) irakiensis, P. (P.) tunguidus, P. (P.) blanci, and P. (P.) longipes. The right P5Exp-2 of the new species has a principal lateral spine on the proximal half of the outer margin, but on the middle length in P. (P.) irakiensis and on the distal half in P. (P.) blanci and P. (P.) tunguidus. The new species’ left P5 has a two-segmented Enp but an unsegmented Enp in P. (P.) blanci and P. (P.) longipes. The new species has untransformed caudal rami, versus transformed ones in P. (P.) tunguidus. The urosomites 2 and 3 of the new species lack hairs, but urosomite 2 is hairy in P. (P.) blanci, and both somites are hairy in P. (P.) longipes. Segment XX of the right antennule of the new species has a serrated process versus a smooth one in P. (P.) longipes. The female of the new species has only one right spine on the genital double-somite, versus a pair of spines in other species. Moreover, the new species lacks spines on the P5Exp-2 but has outstanding spines in its congeners (see Table 4).

The new species (Fig. 6A) is most similar to P. (P.) christineae (Fig. 6B), particularly the morphology of the male right P5. The male P5 of the new species has a moderate spine on the right coxa, a long and narrow lamella on the left basis, the right Enp is unsegmented, and there are two spines on the Exp-2 outer margin, whereas the female P5 has an asymmetrical leg and a two-segmented Enp. Other shared characteristics are the right antennule with a serrated spine on segment 20, and the caudal rami are not transformed in males. The new species was easily misidentified as P. (P.) christineae (see discussion below). After re-examination of several samples collected from the Mun River Basin, many major morphological differences are defined in both sexes. The male urosomite of the new species has no ornamentation, versus the hairy urosomite 2 in P. (P.) christineae. There are many different traits in the male P5. The new species has a distal margin of the intersclerite plate that produces a rounded lobe versus the mostly triangular-lobed P. (P.) christineae. The new species has a semi-circular lamella on the right basis, but a long, narrow lamella in P. (P.) christineae; the right Exp-2 is strongly oval in the new species versus round or slightly oval in P. (P.) christineae; the new species has a reduced distal accessory spine on the right Exp-2, which is obviously smaller than those of *P.christineae*; and the new species has a sturdy principal lateral spine inserted at the proximal half of the right Exp-2 outer margin versus slender and presented at the middle of the right outer margin in P. (P.) christineae.

Other differences in the male left P5 are: (1) the new species has two-segmented Enp but is unsegmented in P. (P.) christineae; (2) the new species has no hyaline lamella on Enp but is presented in P. (P.) christineae. However, such hyaline lamellae are hardly observed under a light microscope in some populations of P. (P.) christineae, too (personal observation).

The female of the new species differs from P. (P.) christineae by: (1) having only one spine on the left wing of pediger 5, whereas P. (P.) christineae has a spine on both wings; (2) the new species has only one right spine on the genital double-somite, whereas P. (P.) christineae has a pair of spines; (3) the new species has a conveyor canal along the anterior view of the left P5Exp-2, but this canal is absent in P. (P.) christineae; (4) the female legs of the new species show an apomorphies character for the genus by the Exp-2 without a distal lateral spine, versus P. (P.) christineae, which has a normal form like its congeners by having this spine.

The presence of a spine on the P5Exp-2 in female diaptomid copepods in Thailand is found in the genera *Tropodiaptomus* Kiefer, 1932; *Heliodiaptomus* Kiefer, 1932; *Neodiaptomus* Kiefer, 1932; *Allodiaptomus* Kiefer, 1936; and *Mongolodiaptomus* Kiefer, 1937; but it is the opposite scenario in *Eodiaptomus* Kiefer, 1932, including *E.draconisignivomi* Brehm, 1952; *E.phuphanensis* Sanoamuang, 2001a; *E.phuvongi* Sanoamuang & Sivongxay, 2004; and *E.sanuamuangae* Ranga Reddy & Dumont, 1998. The genus *Eodiaptomus* has only two species with such a spine on P5Exp-2, i.e., *E.japonicus* (Burckhardt, 1913) from Japan and *E.sinensis* (Burckhardt, 1913) from China, which perhaps use the reduced form or absence of this lateral spine as a generic character (Ranga Reddy and Dumont 1998). In addition, the genus *Dentodiaptomus* Shen & Tai, 1964, also shared this character with the new species, which is found in the recent species described from Thailand, *D.orientalis* Sanoamuang & Watiroyram, 2021. The sharing of synapomorphies among different genera probably reveals their evolutionary relationships, or, in other words, ‘the relationship is based on the synapomorphies possession’ (Boxshall 1986; Sanoamuang and Watiroyram 2021).

In Thailand, the most widespread diaptomid species is P. (C.) praedictus praedictus. It lives in both temporary and permanent water bodies. During the rainy season, it was also discovered in seeping pools in caves as a result of drift from the surface (Watiroyram 2021). After P. (C.) praedictus praedictus, P. (P.) christineae is the most often encountered species, followed by P. (P.) thailandicus, which is likewise found in both ephemeral and permanent water bodies (Sanoamuang 2002). Conversely, P. (P.) roietensis and P. (P.) surinensis are very rare and restricted to temporary water bodies (Sanoamuang 2002; Sanoamuang and Watiroyram 2020; Sanoamuang and Dabseepai 2021). Phyllodiaptomus (P.) parachristineaesp. nov. was present in both temporary and permanent water bodies from the Mun River basin in the northeastern part of Thailand and was found more often than P. (P.) roietensis and P. (P.) surinensis, respectively. The distribution of P. (P.) christineae and P. (P.) parachristineaesp. nov. is puzzling when reviewing unpublished documents from previous studies. Prior authors probably identified the new species as its closet species, P. (P.) christineae, especially the specimens collected from the Chi River and Mun River basins. Based on the available information, P. (P.) christineae lives mostly in large water bodies, like lakes, rivers, and reservoirs, in the north, center, and east of Thailand, and it is rare in the Songkhram River basin. P. (P.) parachristineaesp. nov., on the other hand, lives in small water bodies rather than large ones in the Mun River basin from lower northeastern Thailand to Cambodia. However, more sampling and specimen examinations are required to explain their distribution in the northeast of Thailand.

The distribution and habitats of the 13 species and two subspecies of *Phyllodiaptomus* in Asia are represented in Table 5. The common species live in a wide variety of habitats from small to large water bodies, such as P. (P.) blanci, which has been reported in Iran, Iraq, Israel, India, Nepal, Uzbekistan, Kazakhstan, Turkmenistan, and Tajikistan. Although P. (C.) praedictus praedictus and P. (P.) parachristineaesp. nov. have been found in both temporary and permanent water bodies, they have so far been known only from Thailand and Cambodia. Species that live in large water bodies (i.e., ponds, lakes, or rivers) also show a widespread distribution, such as P. (C.) annae (Sri Lanka, India, Bangladesh), P. (C.) praedictus sulawesensis (Indonesia, Philippines), and P. (P.) christineae (Thailand, Laos), except P. (P.) irakiensis and P. (P.) longipes, which are exclusively found only in Iraq and Indonesia, respectively. The small water bodies or temporary waters are separated from other waters, and then it is a naturally occurring barrier for species distribution, such as in the cases of rare species or endemic species like P. (C.) sasikumari (India), P. (P.) roietensis (Thailand, Cambodia), and P. (P.) surinensis (Thailand) (Ranga Reddy 1994; Sanoamuang 1999; Khalaf 2008; Alekseev et al. 2013; Marrone et al. 2014; Kulkarni and Pai 2016; Guinto et al. 2018; Sanoamuang and Watiroyram 2020; Sanoamuang and Dabseepai 2021) (see Table 5).

**Table 5.** Distribution and habitats of the 13 species and two subspecies of the genus *Phyllodiaptomus* in Asia.

No.	Species	Distribution	Habitats	References
**Subgenus Phyllodiaptomus (Ctenodiaptomus)Dumont et al., 1996**				
1	P. (C.) annae (Apstein, 1907)	Sri Lanka, India, Bangladesh	Lake, pond	Ranga Reddy (1994)
2	P. (C.) praedictus praedictus Dumont & Ranga Reddy, 1994	Thailand, Laos, Cambodia	Roadside canal, ricefield, pond, swamp, lake, reservoir and river	Sanoamuang (1999); Sanoamuang and Dabseepai (2021)
	P. (C.) praedictus sulawesensis Alekseev & Vaillant, 2013	Indonesia, Philippines	Lake and swamp	Alekseev et al. (2013); Guinto et al. (2018)
3	P. (C.) sasikumari Ranga Reddy & Venkateswarlu, 1989	India	Pool, paddy field and ephemeral pond	Ranga Reddy (1994)
4	P. (C.) wellekensae Dumont & Ranga Reddy, 1993	India	Pool and ephemeral pond	Ranga Reddy (1994)
**Subgenus Phyllodiaptomus (Phyllodiaptomus)Dumont et al., 1996**				
5	P. (P.) blanci (Guerner & Richard, 1896)	Iran, Iraq, Israel, India, Nepal, Central Asia (Uzbekistan, Kazakhstan, Turkmenistan, Tajikistan)	Shallow and large water bodies	Ranga Reddy (1994); Marrone et al. (2014); Kulkarni and Pai (2016)
6	P. (P.) christineae Dumont, Ranga Reddy & Sanoamuang, 1996	Thailand, Laos	Irrigation canal, pond, lake, river and reservoir	Sanoamuang (1999); Sanoamuang and Dabseepai (2021)
7	P. (P.) irakiensis Khalaf, 2008	Iraq	River	Khalaf (2008)
8	P. (P.) longipes Kiefer, 1965	Indonesia	Lake	Ranga Reddy (1994)
9	P. (P.) parachristineaesp. nov.	Thailand, Cambodia	Roadside canal, ricefield, pond and reservoir	Sanoamuang and Dabseepai (2021); Chaicharoen and Sanoamuang (2022); This study
10	P. (P.) roietensis Sanoamuang & Watiroyram, 2020	Thailand, Cambodia	Roadside canal and ephemeral pond	Sanoamuang and Watiroyram (2020)
11	P. (P.) surinensis Sanoamuang & Yindee, 2001	Thailand	Irrigation canal	Sanoamuang and Yindee (2001)
12	P. (P.) thailandicus Sanoamuang & Teeramaethee, 2006	Thailand	Shallow water bodies	Sanoamuang and Teeramaethee (2006); Sanoamuang and Dabseepai (2021)
13	P. (P.) tunguidus Shen & Tai, 1964	China, Laos, Vietnam	River, lake, pond, reservoir	Boonmak and Sanoamuang (2022); Zhang et al. (2021, 2023)

## Supplementary Material

XML Treatment for Phyllodiaptomus (P.) parachristineae

## ﻿Additional information

### Author ORCIDs

Laorsri Sanoamuang https://orcid.org/0000-0003-0377-1225

Santi Watiroyram https://orcid.org/0000-0003-4920-3979

### Data availability

All of the data that support the findings of this study are available in the main text.
